# Strength of primary care service delivery: a comparative study of European countries, Australia, New Zealand, and Canada

**DOI:** 10.1017/S1463423617000792

**Published:** 2018-01-08

**Authors:** Danica R. Pavlič, Maja Sever, Zalika Klemenc-Ketiš, Igor Švab, Milena Vainieri, Chiara Seghieri, Alem Maksuti

**Affiliations:** 1 Department of Family Medicine, Medical Faculty, University of Ljubljana, Ljubljana, Slovenia; 2 Statistical Office of the Republic of Slovenia, Ljubljana, Slovenia; 3 Department of Family Medicine, Medical Faculty, University of Maribor, Maribor, Slovenia; 4 Community Health Center Ljubljana, Ljubljana, Slovenia; 5 Laboratory of Management and Health, Institute of Management, Sant’Anna School of Advanced Studies, Pisa, Italy; 6 Institute for Political Management, Ljubljana, Slovenia

**Keywords:** comparative method, cross-country study, healthcare expenditure, primary care, strength

## Abstract

**Aim:**

We sought to examine strength of primary care service delivery as measured by selected process indicators by general practitioners from 31 European countries plus Australia, Canada, and New Zealand. We explored the relation between strength of service delivery and healthcare expenditures.

**Background:**

The strength of a country’s primary care is determined by the degree of development of a combination of core primary care dimensions in the context of its healthcare system. This study analyses the strength of service delivery in primary care as measured through process indicators in 31 European countries plus Australia, New Zealand, and Canada.

**Methods:**

A comparative cross-sectional study design was applied using the QUALICOPC GP database. Data on the strength of primary healthcare were collected using a standardized GP questionnaire, which included 60 questions divided into 10 dimensions related to process, structure, and outcomes. A total of 6734 general practitioners participated. Data on healthcare expenditure were obtained from World Bank statistics. We conducted a correlation analysis to analyse the relationship between strength and healthcare expenditures.

**Findings:**

Our findings show that the strength of service delivery parameters is less than optimal in some countries, and there are substantial variations among countries. Continuity and comprehensiveness of care are significantly positively related to national healthcare expenditures; however, coordination of care is not.

## Introduction

Primary care (PC) is established as the foundation for the population’s healthcare in a large part of the world. PC provides a consistent point of care over the long term, tailoring, and coordinating care for those with multiple healthcare needs and supporting patients in self-education and self-management (Organisation for Economic Co-operation and Development (OECD), 2014). Because the majority of chronically ill patients receive the bulk of their care through PC, coordination of care across the primary, secondary, and tertiary levels is of critical importance (Anderson *et al*., 2003). PC can be conceived of as a sub-system of the overall healthcare system, with a special focus on facilitating access to and utilization of coordinated services for the benefit of a population’s health (Kringos *et al*., 2015: 33). Although the World Health Organization (WHO, 1978) recommended rethinking care-delivery models by improving and strengthening the role of PC, there are still significant differences among them, ranging from the structure to the processes and outcomes of care.

The strength of a country’s PC system is determined by the degree of development of a combination of core PC dimensions in the context of its healthcare system (Kringos *et al*., 2010: 2). There is no universal recipe for creating and developing a strong PC system.

According to an extensive literature review, Kringos *et al*. (2010) recognize the lack of detail in documents that constitute a strong and effective PC system. They also highlight that little attention has been paid to systematic PC development. Although strengthening healthcare systems has been at the centre of activities in many countries for more than a decade (Boerma and Dubois, 2006), there are only a few holistic and overall ratings or tools that allow a comparison of PC systems in terms of service delivery (Detollenaere *et al*., 2017).

Based on the results of a systematic review (Kringos *et al*., 2015), PC has been unravelled into essential ingredients, called dimensions, which have been ordered into three groups: those related to the structure, the process, and the outcome of care, respectively. The structure dimension refers to the basic conditions that enable good functioning of PC, consisting of relevant policies and regulations as well as the availability of financial, human, and material resources. The process of PC includes dimensions relevant to the services that are delivered (access, continuity, coordination, and comprehensiveness). A core outcome is improved health of the population, but efficiency and equity are also considered as such (Kringos *et al*., 2015: 33).

A WHO resolution adopted few years ago (A62/8) urges member states to strengthen their healthcare systems through the values and principles of primary health (WHO, 2009). Considering the specifics of a country’s PC systems, potential improvements rely on the alignment of the various elements of PC systems (ie, healthcare expenditure systems), the health workforce, technologies and the information system, leadership and governance, and service delivery, which special attention is paid to in this study.

This study examines the relationship between the strength of PC service delivery and healthcare expenditure in each country. Our analysis seeks to answer the following questions: to what extent does the strength of PC process service delivery and available healthcare resources vary among countries? Does a country’s position on an overall healthcare expenditure dimension reflect its performance on the strength of PC service delivery? Can countries be meaningfully grouped based on their levels of healthcare expenditure and strength of PC processes?

## Methods

### Setting

A comparative cross-sectional study design was applied using the Quality and Costs of Primary Care in Europe (QUALICOPC) GP database. The QUALICOPC project was a set of four surveys designed to collect information about the practice setting, services provided, patient values, and patient experience. Our study presents the results for GPs. Details about the study protocol and questionnaire development have been published elsewhere (Schäfer *et al*., 2011; 2013).

The QUALICOPC study was conducted among GPs and patients in 31 European countries (the EU-27 except France, plus Macedonia, Iceland, Norway, Switzerland, and Turkey) and three non-European countries (Australia, Canada, and New Zealand). Data collection took place from October 2011 to December 2013. QUALICOPC was a European Commission-funded project under the Seventh Framework Programme.

### Participants

In each country, a nationally representative sample of GPs and patients completed standardized questionnaires. In Turkey, Spain, Belgium, and Canada, larger samples were taken in order to make comparisons between regions (Wong *et al*., 2015). The actual number of GPs approached varied between countries. The sampling characteristics and response rates of QUALICOPC GPs studied by country are detailed in Table 1.

**Table 1:** Sampling characteristics with response rates of GPs studied by country (*n*=6734)

Country	Sampling procedure^a^	Recruitment method	GPs invited	GPs participated	Response rate (%)
Europe					
Austria	B	Email, personal	3050	173	6
Belgium	B	Letter, telephone, email	5000	382	8
Bulgaria	B	Telephone, face-to-face	350	209	60
Cyprus	A	Letter, telephone	90	67	74
Czech Republic	B	Letter, telephone, personal contact	520	205	39
Denmark	B	Email	2000	199	10
England	C	Letter, email	1508	160	11
Estonia	A	Letter, telephone, email	802	121	15
Finland	D	Letter, email, telephone, personal contact	1000	270	27
Germany	B	Letter	3825	223	6
Greece	D	Telephone, letter	300	206	69
Hungary	B	Email and personal contact	400	209	52
Iceland	A	Letter and personal contact	95	75	79
Ireland	D	Letter, email, personal contact, advertisement	2515	158	6
Italy	E	Telephone	Unknown	204	Unknown
Latvia	B	Telephone, email	545	205	38
Lithuania	B	Personal, telephone	508	211	42
Luxembourg	A	Telephone	120	73	61
Macedonia	B	Letter, email	240	134	56
Malta	B	Telephone	78	65	83
The Netherlands	B	Letter, email, telephone	1400	224	16
Norway	E	Letters, telephone, conferences	500	185	37
Poland	C	Letter, telephone, email	665	206	31
Portugal	B	Letter, email, telephone	800	203	25
Romania	B	Letter, telephone, email, personal contact	399	206	52
Spain	C	Email, telephone	500	402	80
Slovakia	B	Letter, telephone, personal contact	1000	206	21
Slovenia	B	Letter, telephone, email	1173	194	17
Sweden	B	Letter	1000	91	9
Switzerland	B	Letter, telephone	2027	186	9
Turkey	C	Letter and personal contact	1300	281	22
Outside Europe					
Australia	D	Letter	3201	142	4
Canada	B	Letter, email, telephone	23 671	502	2
New Zealand	B	Letter	1371	157	11

a Sampling procedures codes: A=(almost) entire GP population; B=random national sample (stratified or not); C=random sample in preselected regions; D=mixed procedure (random procedure plus selected GPs); E=‘opportunity sampling’/volunteers.

The final sample included 6734 GPs from 34 different countries. The average response rate was 32.6% and ranged from 2% in Canada to 83% in Malta (Table 1).

### Questionnaire, indicators, and scales

Data on the strength of PC were collected using a standardized GP questionnaire, which included 60 questions divided into 10 dimensions related to process (access, continuity, coordination, comprehensiveness), structure (governance, economic conditions, and workforce development), and outcomes (eg, correlation between PC system strength, and healthcare expenditure), which together illustrate the framework for assessing the strength of a particular national PC system. The GP questionnaire was completed either on paper or electronically (online or using a tablet computer). Trained research units in each participating country were in charge of coordinating the data collection.

In our study, we selected 13 questions from the GP questionnaire that are intended to summarize the strength of PC process service delivery in terms of the following three dimensions: continuity, coordination, and comprehensiveness of care. The three process dimensions were measured through composites/scales, in which multilevel analyses are used to construct scale scores. In this methodology, scale items are used as the lower level of the model and nested for the individual that provided the answers to the questions. The residuals of each level are used to construct scale scores at different levels (patient, GP, and country) (Leyland and Groenewegen, 2003; Kringos *et al*., 2010).

Because indicators on service delivery have different metrics (ranges), they were expressed as *z*-scores. Their composites were calculated as a sum of *z*-scored indicators. We analyzed an individual indicator in the sense of a positive or negative assessment by GPs. In doing so, we took into account the appertaining value interval and the average value calculated. As an example, the indicator ‘mr1’ was defined with an interval of 0–1, and its average value was 0.892. The threshold value for determining the positive/negative orientation of the assessment is found at the mean of the interval, which in this particular case is 0.5. This means that, on average, GPs assessed the indicator positively (mr1 average value was 0.892). This method was used to evaluate all 13 indicators. A negative orientation of a country’s composite score indicates a below-average position, whereas a positive orientation points to an above-average position. The service delivery indicators used in the analysis and divided into the three dimensions are shown in Table 2.

**Table 2:** Selected indicators on the strength of primary care process service delivery, by dimension

Dimension	Indicator	Code	Range
Continuity of care	Medical recordkeeping: inclusion of important information	mr1	0–1
	Medical recordkeeping: regularity of keeping	mr2	0–1
	Informational continuity of care with PC	infpc	1–3
	Informational continuity of care with secondary care	infsc	1–5
Coordination of care	Skill mix: disciplines working in practice	skill	0–1
	Integration of primary and secondary care: information from medical specialists	pcsc	1–3
	Collaboration with other providers: meeting face-to-face with other professionals	collab	1–3
	Community orientation: reporting certain occurrences	comor	1–4
Comprehensiveness of care	Medical equipment available	mea	0–1
	First contact for common health problems	fcgp	1–4
	Treatment and follow-up of patients with diagnoses	treat	1–4
	Medical technical procedures and preventive care	tech	1–4
	Health promotion activities	prom	0–1

PC=primary care.

In order to examine the relationship between the country strength of PC process service delivery and healthcare expenditures, we selected the World Bank indicator on country healthcare expenditure per capita purchasing power parity (PPP). The data are for 2012, or for the period in which the QUALICOPC study was carried out (The World Bank, 2015).

### Statistical analysis

Descriptive statistics were used to analyse sampling characteristics, selected indicators, and composites. Pearson’s correlation coefficients were used to evaluate the relationships between process indicators and healthcare expenditures. For interpreting the correlation, we defined the following:<0.20=very low, 0.20–0.40=low, 0.40–0.60=moderate, 0.60–0.80=high, and 0.80–1.00=very high. The confidence level was set at *P*⩽0.05. Analyses were conducted using IBM SPSS Statistics for Windows (version 22; SPSS Inc., Chicago, IL, USA).

The average ranks were calculated as follows: countries were first ranked by the value of an individual indicator and then numbered consecutively (1 represented the best position in the context of an individual indicator). If more than one country had the same indicator value, they received the same rank. Then, based on this classification, we calculated an average, on the basis of which we once again re-ordered the countries. This final calculation was defined as the average rank value.

## Results

### Service delivery assessment

Table 3 shows the results of GPs’ evaluation scores on the strength of PC process service delivery indicators and composites by country.

**Table 3:** GPs’ evaluation scores on process service delivery indicators and composites, by country

	Continuity of care						Coordination of care						Comprehensiveness of care						
Country	mr1	mr2	infpc	infsc	Average rank	Composite score	skill	pcsc	collab	comor	Average rank	Composite score	mea	fcgp	treat	tech	prom	Average rank	Composite score
Europe																			
Austria	0.874	0.954	1.751	3.923	20	−0.556	0.055	1.720	1.711	3.318	21	−1.331	0.586	2.773	3.329	2.070	0.305	16	0.618
Belgium	0.910	0.845	2.591	4.095	14	0.732	0.021	1.708	1.880	3.271	23	−1.035	0.418	3.017	3.344	2.372	0.172	17	0.045
Bulgaria	0.776	0.696	1.990	3.942	24	−1.296	0.056	1.944	2.077	3.310	13	0.414	0.364	2.966	3.154	1.764	0.216	20	−1.143
Cyprus	0.863	0.769	1.591	2.250	27	−2.533	0.474	1.538	2.058	2.548	20	−0.105	0.365	2.089	2.835	1.248	0.236	27	−4.103
Czech Republic	0.926	0.772	2.971	4.083	12	1.149	0.040	1.663	1.830	3.252	24	−1.249	0.293	2.431	2.711	1.392	0.232	27	−3.832
Denmark	0.896	0.769	2.949	4.402	12	1.144	0.093	1.637	1.561	3.248	25	−1.988	0.750	3.375	3.545	2.532	0.069	12	2.116
England	0.985	0.974	2.968	4.154	5	2.077	0.464	1.684	2.074	3.397	9	1.877	0.604	3.275	3.568	2.633	0.402	7	3.493
Estonia	0.856	0.456	2.545	4.083	22	−0.688	0.130	1.607	1.962	2.619	23	−1.625	0.505	2.730	3.210	1.534	0.272	19	−0.919
Finland	0.905	0.741	2.776	3.646	19	0.300	0.643	1.918	2.095	3.310	7	3.192	0.884	2.735	3.261	3.308	0.076	15	1.853
Germany	0.945	0.986	2.788	4.037	9	1.527	0.046	1.805	1.587	3.073	25	−2.061	0.647	2.822	3.470	1.832	0.363	13	1.326
Greece	0.758	0.281	1.727	2.359	32	−3.803	0.198	1.727	2.086	3.466	9	0.857	0.527	2.685	3.014	2.415	0.343	18	0.225
Hungary	0.892	0.728	2.375	4.408	18	0.250	0.064	1.454	2.037	2.865	25	−1.545	0.360	2.697	3.363	1.372	0.152	24	−2.224
Iceland	0.757	0.932	2.863	3.197	20	−0.420	0.442	1.930	2.073	3.390	7	2.312	0.819	2.689	2.977	2.877	0.092	19	0.424
Ireland	0.892	0.981	2.777	3.647	15	0.776	0.246	1.483	1.985	3.249	20	−0.138	0.623	3.251	3.593	2.670	0.199	9	2.404
Italy	0.900	0.495	1.436	4.391	21	−1.410	0.032	1.573	1.700	3.454	23	−1.582	0.193	2.866	3.375	1.392	0.069	25	−3.033
Latvia	0.912	0.655	2.420	4.015	19	0.049	0.144	1.859	2.073	3.095	16	0.215	0.469	2.639	3.216	1.429	0.157	24	−2.074
Lithuania	0.843	0.479	2.910	3.900	21	−0.311	0.693	1.677	2.059	3.309	12	2.765	0.731	3.107	2.942	1.270	0.305	17	0.095
Luxembourg	0.961	0.932	2.137	3.514	18	0.158	0.033	1.726	1.681	3.268	24	−1.561	0.431	2.693	3.113	2.075	0.158	22	−1.472
Macedonia	0.853	0.722	1.850	4.241	21	−0.954	0.057	1.718	1.898	3.328	17	−0.651	0.241	3.057	3.365	1.275	0.205	21	−1.762
Malta	0.757	0.588	1.531	3.000	31	−3.521	0.227	1.548	1.915	3.329	18	−0.199	0.474	2.815	2.857	2.188	0.060	24	−2.051
The Netherlands	0.878	0.987	2.996	3.898	11	1.209	0.310	2.079	2.129	3.421	4	2.238	0.678	3.357	3.373	3.287	0.149	10	2.826
Norway	0.938	0.978	2.541	4.044	12	1.082	0.107	2.018	1.853	3.590	12	0.491	0.842	3.221	3.510	3.131	0.113	10	3.130
Poland	0.848	0.930	1.966	3.107	24	−1.126	0.310	1.502	2.094	3.311	15	0.723	0.477	2.637	3.194	1.318	0.073	27	−2.729
Portugal	0.965	0.886	2.374	4.167	13	0.938	0.221	1.351	1.973	3.106	24	−0.834	0.332	3.064	3.313	1.804	0.165	20	−1.065
Romania	0.906	0.610	2.442	3.897	22	−0.290	0.054	1.595	1.882	3.294	23	−1.048	0.227	2.880	3.303	1.426	0.118	25	−2.660
Spain	0.970	0.463	2.737	4.444	13	0.672	0.557	1.478	1.736	3.415	17	0.758	0.693	3.144	3.494	2.204	0.227	11	1.856
Slovakia	0.673	0.380	2.510	3.925	26	−1.899	0.044	1.630	1.809	3.334	22	−1.221	0.245	2.598	2.616	1.298	0.252	27	−3.931
Slovenia	0.930	0.782	2.985	3.827	14	0.998	0.322	1.554	2.025	3.331	15	0.664	0.565	2.994	3.655	1.448	0.361	12	1.482
Sweden	0.982	0.967	2.600	3.846	13	1.298	0.500	1.673	2.122	3.134	13	1.730	0.862	3.402	3.574	3.009	0.127	7	3.644
Switzerland	0.984	0.871	2.833	3.966	11	1.496	0.044	1.876	1.716	3.259	22	−1.195	0.846	2.908	3.455	2.589	0.232	11	2.451
Turkey	0.711	0.807	1.317	1.404	30	−4.703	0.126	1.453	1.803	3.553	21	−0.867	0.415	2.409	2.746	1.755	0.084	28	−3.742
Outside Europe																			
Australia	0.975	0.950	2.688	4.072	11	1.510	0.280	1.616	1.829	3.197	22	−0.313	0.639	3.078	3.472	2.881	0.138	13	1.765
Canada	0.952	0.972	2.689	3.549	14	0.958	0.225	1.936	1.970	3.222	15	0.626	0.343	3.118	3.544	2.643	0.173	14	0.664
New Zealand	0.988	0.955	2.987	4.581	3	2.420	0.310	1.703	1.898	3.350	14	0.519	0.701	3.186	3.608	3.207	0.263	6	3.681
Total sample	0.892	0.788	2.471	3.796	–	–	0.222	1.696	1.910	3.281	–	–	0.524	2.926	3.294	2.132	0.193	–	–

Countries were ranked by raw values for each indicator. Then the average rank value was calculated for each dimension.

Eight indicators had a positive assessment and five indicators had a negative valuation. Continuity of care is perceived as the most important dimension regarding the strength of PC process service delivery; however, GPs recognize certain drawbacks in the coordination and comprehensiveness of care settings. Medical technical procedures, preventive care, and health promotion are activities that vary between countries. Coordination of care was identified as the weakest part of the strength in national PC systems. The results (Table 3) show that there are variations in the service delivery among countries both in terms of individual indicators as well as in terms of dimensions.

### Continuity of care

Considering average rank values for continuity of care, New Zealand, England, and Germany have the highest continuity of care in the view of their GPs. Australia is in fourth place together with Switzerland and the Netherlands (all three have an average value of 11), and Czech Republic shares fifth place with Denmark and Norway (all three have an average value of 14). Taking into account composite continuity of care, Turkey, Greece, Malta, Cyprus, and Slovakia are the bottom five countries, where continuity of care lags far behind continuity in other countries (all below −1.5; Table 3).

### Coordination of care

The average rank value for the coordination of care dimension is the highest in the Netherlands, Finland, Iceland, Greece, and England. From a comparative perspective, the lowest status for continuity of care was found in Germany, Denmark, Hungary, the Czech Republic, Luxembourg, and Portugal. New Zealand (with an average rank value of 14) was in sixth place, Canada, Slovenia, and Poland (all three have an average rank value of 15) shared seventh place, and Australia, Slovakia, and Switzerland (all three have an average rank value of 22) shared 13th place. According to the composite scores, Finland, Lithuania, Iceland, the Netherlands, England, and Sweden are the top countries (all above 1). Overall, 19 countries scored below average, including Germany, Denmark, Estonia, Italy, Luxembourg, and Hungary (see Table 3).

### Comprehensiveness of care

In general, health promotion proved to be the weakest part of the comprehensiveness aspect because all 34 countries had negative assessment scores (ie, below the midpoint of the scale). However, health promotion is relatively best arranged in England, Germany, Slovenia, Greece, and Austria, whereas GPs in Malta, Italy, Denmark, Poland, and Finland do not make use of health promotion to a great extent. The composite score perspective shows that comprehensiveness of care is arranged well in New Zealand, Sweden, England, Norway, and the Netherlands (all above 2.5), whereas Cyprus (below −4) was in last place. Regarding average rank, Australia (an average rank value of 13; seventh place), and Canada (an average rank value of 14; eighth place) ranked among the top 10 countries (see Table 3).

### Healthcare expenditure and strength of PC process service delivery

On average, the countries analyzed registered $3116 in healthcare expenditures per capita (PPP) with a wide range: there are countries that spend less than one-third of the average (Macedonia, Romania, and Turkey) and others that spend almost twice the average (Luxemburg and Norway) (Table 4).

**Table 4:** Healthcare expenditure per capita in PPP by country in 2012

Country	Healthcare expenditure per capita in US$ (PPP) 2012
Europe	
Austria	4812.308
Belgium	4345.446
Bulgaria	1171.053
Cyprus	2240.136
Czech Republic	2037.664
Denmark	4615.452
England	3234.717
Estonia	1415.654
Finland	3497.034
Germany	4634.897
Greece	2354.837
Hungary	1765.543
Iceland	3484.906
Ireland	3817.382
Italy	3153.184
Latvia	1256.065
Lithuania	1582.844
Luxembourg	6379.496
Macedonia	796.934
Malta	2522.422
The Netherlands	5394.775
Norway	6059.928
Poland	1509.189
Portugal	2522.422
Romania	982.194
Spain	2925.310
Slovakia	2064.814
Slovenia	2617.696
Sweden	4041.083
Switzerland	5992.449
Turkey	970.597
Outside Europe	
Australia	3855.107
Canada	4609.731
New Zealand	3290.842

Source: The World Bank. Data on expenditures apply to the base year 2012. PPP = purchasing power parity.

There is a significant relationship between country healthcare expenditure and the strength of PC process service delivery (Table 5).

**Table 5:** Estimated correlations^a^ between PHC system strength indicators and healthcare expenditure indicators

Dimension	Indicator	Healthcare expenditure per capita in PPP
Continuity of care	mr1	0.477**
	mr2	0.563**
	infpc	0.334
	infsc	0.200
	Composite score	0.510**
Coordination of care	skill	−0.067
	pcsc	0.458**
	collab	−0.378*
	comor	0.197
	Composite score	−0.014
Comprehensiveness of care	mea	0.531**
	fcgp	0.400*
	treat	0.442**
	tech	0.677**
	prom	−0.040
	Composite score	0.620**

a Pearson correlation coefficients.

*Significant at 0.05; **Significant at 0.01. PHC = primary healthcare; PPP = purchasing power parity.

**Figure 1:**
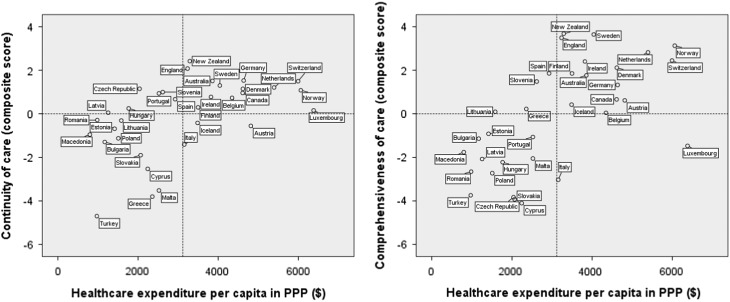
Continuity and comprehensiveness of care versus healthcare expenditure indicators. For ease of presentation, the reference line on the *y*-axis was added at 0. Countries above the reference line have positive (above-average) evaluation scores, whereas those below the line have negative (below-average) scores.

Total healthcare expenditure per capita in PPP in 2012 significantly correlated with composite continuity and comprehensiveness of care, but not with coordination of care (Table 4). The positive correlation is more evident on plots with total healthcare expenditure per capita in PPP. The majority of countries can be found in either the bottom left or upper right quadrants, indicating that countries with higher total healthcare expenditures per capita in PPP also tend to have a higher continuity and comprehensiveness of care (Figure 1).

## Discussion

Our study showed that there were considerable variations in PC service delivery and in healthcare expenditures across the countries studied. Continuity and comprehensiveness of care – as the elements of PC process – proved to be significantly linked to healthcare expenditure settings, whereas coordination of care did not.

The results of our study showed that PC systems in the studied countries differ according to the strength of PC process service delivery. The countries outside Europe (Australia, Canada, and New Zealand) perform better in terms of strength of PC process service delivery than those in Europe (Table 3). The strength of PC process service delivery situation is quite diverse in European countries. GPs outside Europe perceived the coordination of care as the weakest and most problematic area. PC systems in Cyprus, Malta, Slovakia, and Turkey appeared to be ‘weak’ because they obtained repetitive negative composite scores in all three ‘process service delivery’ areas (continuity, coordination, and comprehensiveness). Considering the sum of average ranks for all three process service delivery dimensions observed (fewer points means a better ranking), the best overall PC process service delivery in Europe is observed in England (the sum of the average rank values is 21) and the Netherlands (the sum of the average rank values is 25), whereas among non-European countries the best overall PC process service delivery is shown by New Zealand, which took second place (the sum of the average rank values is 23 points). Canada (the sum of the average rank values is 43) ranked ninth, and Australia shared 12th with Iceland, scoring the sum of the average rank values of 46. The result may be due to successful reforms in Australia, England, and the Netherlands, and policies that promote quality at the primary level (Willcox *et al*., 2011; Nicholson *et al*., 2012).

The challenge of comparing the process service delivery of PC systems and healthcare expenditures is tricky. However, the results of our study showed that total healthcare expenditure per capita in PPP correlated significantly with two PC process service delivery dimensions: comprehensiveness and continuity of care. Pelone *et al*. (2013) showed that investments in economic conditions were very important in order to achieve an efficient structure-process balance in efficiency, which in turn affects the strength of PC process service delivery. This was also proved in our study.

We consider that the coordination of care relates most strongly to planners of PC and the opportunities offered by health managers in the local community. Factors, that among others, may influence the coordination of care, are awareness of relational coordination by organizational leaders, shared goals, frequency and timeliness of communication, transfer of information, negotiation of responsibilities, and problem solving communication (Schultz *et al*., 2013; Rundall *et al*., 2016; Van Houdt *et al*., 2014). On the other hand, medical equipment, technical medical procedures, preventive care, and health promotion activities are related to the amount of healthcare expenditures. The results obtained show that four comprehensiveness items are significantly related to healthcare expenditure; the only exception is health promotion activities. The strongest correlation is demonstrated for medical technical procedures and preventive care (0.677).

Correlations between the strength of PC process service delivery and healthcare expenditure indicator have shown that the strength of PC systems is importantly linked to health expenditure level. However, the continuity aspect of PC proved to be significantly related to health expenditure level and comprehensiveness of care, whereas coordination did not show any significant link to financial resources. The strongest relation proved to exist between health expenditure level and comprehensiveness of care.

Correlations between strength of PC process service delivery parameters and healthcare expenditure at the level of individual countries are rather complex. Luxembourg, for example, has the highest healthcare expenditure per capita in PPP, but its GPs evaluated continuity of care as average and comprehensiveness of care notably below average. Norway, Switzerland, and the Netherlands also have a high level of healthcare expenditure per capita in PPP and a high level of continuity and comprehensiveness of care. On the other hand, Macedonia, Turkey, Romania, Bulgaria, and Latvia have the lowest healthcare expenditure per capita in PPP, and their GPs frequently reported problematic strength indicators. Turkish GPs evaluated continuity and comprehensiveness of care with the most negative scores. Because we carried out the research during the economic crisis in Europe, this might have influenced the results. The classification of specific countries according to strength and financial indicators may partly be a consequence of the crisis. Countries that are frequently found scoring very low are mentioned in other articles as ones with substantial problems in the healthcare sector as a consequence of the economic crisis (Golinowska *et al*., 2006; Catalano, 2009; Notara *et al*., 2010; Karanikolos *et al*., 2013; Kentikelenis *et al*., 2014).

The results of our study also showed that, in general, countries with a better financial position are stronger in PC process service delivery. Looking at continuity/comprehensiveness and healthcare expenditure positions, some country patterns can be recognized. We found that PC systems outside Europe (Australia, Canada, and New Zealand) tend to be most similar to PC systems in England, Denmark, Germany, Ireland, and Sweden. Norway, Switzerland, and the Netherlands can be identified as the countries with strongest PC process service delivery. On the other hand, countries with the weakest PC service delivery do show substantial variability.

This study is consistent with the results of the study conducted by (Kringos *et al*., 2013) in Europe on 2010 data. They found that strong PC systems are linked to better population health but also to higher health spending. On the other hand, although these authors found that cost growth is smoother in countries with more comprehensive PC (Kringos *et al*., 2013), we found that the coordination dimension is an exception that is not correlated with healthcare expenditure.

Economic efficiency should not be the primary goal of any healthcare system and the findings of present study confirm the findings by Kringos *et al*. (2013), that strong PC systems in Europe are linked to better population health but also to higher health spending.

## Conclusions

The results of our study provide a picture of the strength of PC process service delivery in relation to healthcare expenditure per capita in PPP in Europe, Australia, New Zealand, and Canada. We determined that the variation in selected strength indicators among European countries was high, and that some countries were more likely to consistently appear at the top or bottom of the scale. We can confirm that healthcare expenditure is significantly linked to the strength of PC process service delivery. However, the continuity and comprehensiveness dimensions proved to be markedly correlated with healthcare expenditure, but not with the coordination dimension.

The strength of this study lies in its comparative character. The results are based on a pragmatic study design that reflects the presentation and daily management of patients in general practice from a comparative perspective. On the other hand, a limitation of the study is that it compares only the composite indicators of the strength of PC process service delivery and general health expenditure shares. Furthermore, practice activities and characteristics were self-reported by GPs; they are subjective and could therefore be inaccurate; they could be under- or over-reported. The limitations of this study are also related to the fact that the analysis is based only on GPs’ responses. Taking into account the entire sample, GPs had the most uniform opinion on the parameters ‘treatment and follow-up’ (14.7%, standard deviation in %) and ‘collaboration with other providers’ (15.2%). Their answers were very dispersed for ‘skill mix’ (95.5%) and ‘health promotion’ (85.6%). We are aware that individual characteristics of physicians and their clinics have a significant impact on the variability of strength indicators. Therefore we are preparing an additional analysis in which we will assess the impact of the share of individual characteristics.
